# Green synthesis of diazo-pyrazole derivatives and their application as functional disperse dyes for dyeing polyester fabric

**DOI:** 10.1038/s41598-026-51883-3

**Published:** 2026-05-19

**Authors:** Hany Kafafy, Aziza M. Hussien, Galal H. Sayed, Hamada Mashaly, Kurls E. Anwer

**Affiliations:** 1 Dyeing, Printing and Textile Auxiliaries Department, Textile Research and Technology Institute, National Research Centre, 12622-Dokki, Giza, Egypt; 2 Proteinic and Man-made Fibers Department, Textile Research and Technology Institute, National Research Centre, 12622-Dokki, Giza, Egypt; 3 Heterocyclic Synthesis Lab, Chemistry Department, Faculty of Science, Ain Shams University, 11566-Abbassia, Cairo, Egypt

**Keywords:** Pyrazole derivatives, green chemistry, disperse dyes, polyester fabrics, antimicrobial activity, ultraviolet protection, Chemistry, Materials science

## Abstract

PET fibers are widely acclaimed as the most extensively produced synthetic fibers. They are distinguished by low cost, recyclability as well as excellent physical, chemical and mechanical properties. They are used in several applications such as apparel and home furnishings. Pyrazole-based disperse diazo dyes were synthesized using 3-(2-(4-(Phenyldiazenyl)phenyl)hydrazono)pentane-2,4-dione **(1)** as a starting material. Green techniques (grinding and microwave) were used to prepare the new dyes (**2–4)**. The chemical structures and formulae of these dyes were characterized by spectroscopic tools such as IR, ^1^ H-NMR, ^13^C-NMR, and mass spectra. The synthesized dyes were utilized for simultaneous dyeing and functionalization of PET fabric using exhaustion method. Color strength and color fastness of the dyed PET fabrics were examined toward washing, rubbing, perspiration and light. Ultraviolet protection factor (UPF) and antimicrobial activity against *S. aureus* (gram-positive bacteria), *E. coli* (gram-negative bacteria) and *C. albicans* (fungi) were investigated. It was found that the dyed fabrics showed good color fastness properties, excellent UV protection and high antimicrobial activities. These dyed and functionalized fabrics can be used in several applications such as multifunctional garments, upholstery and blankets.

## Introduction

Heterocyclic compounds containing two nitrogen such as pyrazole framework are very important heterocyclic derivatives in both biology and chemistry, so they are usually used in vitamins, natural products, pharmaceuticals, dyes, corrosion, and reagents syntheses^1–7^. It is common that pyrazole derivatives have highly improved therapeutic properties^8–10^. These particular and specific properties have earned the organic chemists to pay attention to the golden key molecule with more different substations which determine the maximum interactions with a DNA or specific protein and determine the biological and pharmaceutical selectivity for the desired molecule^11–13^. Pyrazole derivatives are commonly reported to exhibit antiviral^14^, antibacterial^15^, antimicrobial^16^, anticancer^17^, antagonist^18^, anti-inflammatory^19^, anthelmintic^20^, cardiotonic^21^, herbicidal^22^, CDK-9 inhibitors^23^, analgesic^24^, antimitotic^25^, insecticidal^26^, and antioxidant activities^27^. Furthermore, diazene has high biological reactivity which elucidated that it can be used to synthesize many reactive heterocyclic intermediates used to afford dyes with pharmaceutical properties^28–30^.

Green chemistry is the science of designing chemical substances and processes that minimize or eliminate the use and generation of harmful compounds hence prevent pollution at a molecular level. Various techniques such as grinding and microwave are among green chemistry methods that are used in the synthesis of heterocyclic compounds. Unlike the thermally conventional method, both grinding and microwave irradiation techniques are eco-friendly, easily controlled and more environmentally tolerant. As advantages, several reactions of the heterocyclic derivatives were performed with cleaner and milder conditions, higher yield and shorter reaction time^31–34^.

The solar radiation or UV radiation is divided into three regions (UVA, UVB and UVC). UV radiation that reaches the earth comprises of UVA (320–395 nm) and UVB (320–395 nm). They are responsible for skin aging, wrinkling, skin cancer, and eye damage. While UVC (200–280 nm) is absorbed by the earth’s ozone layer. As a result of ozone depletion, humans become more susceptible to harmful UV radiation. In this concern, the design, production and using of textile materials functionalized with sun protective property is significantly important to reduce the detrimental consequences of UV radiation. UPF is used to estimate the UV protection performance of textiles. Several factors affect the UPF viz. fiber type, fabric construction, dyes, finishes, etc. It is known that many textile dyestuffs absorb sunlight radiation in range of 400–700 nm while others absorb light in the near UV region so dyed textiles often exhibit better UPF^35^.

PET fibers are characterized by low cost, convenient processability and recyclability, and availability for blending with cotton and other natural fibers. They can be produced with controlled morphology and properties. PET fibers have excellent tensile strength, good dimensional and thermal stability as well as resistance to chemicals and mildews. As a result of their distinguished performance, they are used in several textile applications such as garments, sportswear, home furnishings (curtains, carpets, bed sheets, blankets etc.). Moreover, their industrial applications include geotextile, filters, tire cords, ropes, fishing nets, etc.^36,37^.

PET fibers are hydrophobic in nature with low moisture regain and insufficient reactive sites which adversely affect their affinity for dyes. The dyeing of PET fibers carried out with suitable disperse dyes under pressure at temperatures of 120–130°C^38^. Beside the traditional dyeing method^39^, recent green techniques were employed for dyeing and coloration, such as supercritical carbon dioxide^40^, microwave assisted dyeing^41^, atomic layer deposition^42–44^, and D5 non-aqueous media dyeing system^45,46^. These dyeing methods are characterized by short time, salt free and low chemical and water consumption. Azo dyes are widely contributing as disperse dyes in textile industry especially the dyeing of synthetic fabrics. They are distinguished for the wide range of specular and dazzling colors and excellent fastness properties. Researchers have synthesized large numbers of azo dyes containing different moieties to enhance the color strength of the dyed fabrics. PET fabrics were dyed with aryldiazenyl disperse dyes. The dyed fabric has a variety of color shadings ranging from peach amber to apple of my eye depending on the coupler moieties^47^. Monosulfonated azo dyes having auxochromic groups (− OH and −SO_3_H) were used as disperse dyestuffs for dyeing PET. The optimal dyeing conditions were studied in terms of temperature, time, shade and pH. The desired color strength and shades were achieved at 120 °C for 30 min^48^. Moreover, the dyeing behavior of azo disperse dyes containing pyrazole ring was investigated. Three synthetic fabrics, namely, PLA, PET and PA 66 were dyed with a newly synthesized pyrazole disperse dyes and they exhibited yellow-red color shades. The fastness properties of all dyed fabrics were quite high in general. Among the three dyed fabrics, PLA fabrics showed the best sublimation fastness while PET fabrics displayed the highest light fastness performance^49^. Omar et al. prepared new azo pyrazole dyes and examined their dyeing performance on knitted PET fabrics as well as their color fastness. They found that disperse dyes with nitro substituent have the highest color strength (K/S) whereas unsubstituted dyes or that contain chloro substituent showed the lowest K/S. All dyed fabrics exhibited superb fastness properties^50^. Noser et al., have reported the dyeing of polyester fabrics with synthesized pyrazole disperse azo dyes. The dyed fabrics possessed superior ultraviolet (UV) protection as well as antimicrobial activity against *S. aureus*, *B. subtilis*,* P.eruginosa*, *E. Coli*, *C. albicans* and *A. flavus*^5^. Azo dyes based on pyrazolylcarboxamido thiophene derivatives were synthesized and applied for dying PET fabrics. The dyed fabrics showed proper bacterial reduction for both gram-positive (*S. aureus*) and gram-negative (*S. typhimurium*) bacteria^51^. Rizk et al., investigated the antimicrobial effectiveness of PET fabrics dyed with pyrazolotriazine and pyrazolylpyrazolone azo dyes. They mentioned that these dyestuffs have provided fabrics with antimicrobial properties against different types of pathogenic microorganisms^52^.

Herein, this work aims to synthesize diazo-pyrazole derivatives and investigate their application as disperse dyes for dyeing polyethylene terephthalate (PET) fabrics. The synthesis was achieved through the reaction of 3-(2-(4-(phenyldiazenyl)phenyl)hydrazono)pentane-2,4-dione **(1)** as a starting material with hydrazine hydrate, semicarbazide hydrochloride, and thiosemicarbazide to afford the new diazo-pyrazole derivatives **2–4**. Furthermore, a comparison between microwave irradiation and grinding techniques was performed using different green chemistry metrics, including yield economy (YE), atom economy (AE), reaction mass efficiency (RME), and optimum efficiency (OE). The chemical structures of the synthesized compounds were confirmed by elemental analysis and various spectroscopic techniques, including IR, ^1^H-NMR, ^13^C-NMR, and mass spectrometry. In addition, the newly prepared dyes **2–4** were applied to PET fabrics, and their dyeing performance was evaluated in terms of washing, rubbing, perspiration, and light fastness. Moreover, the functional properties of the dyed fabrics, such as antimicrobial activity and UV protection, were also investigated.

## Experimental

### Materials

All starting materials, chemicals, reagents, and solvents were purchased from Sigma-Aldrich with the following purities and catalog numbers: p‑aminoazobenzene (CAS: 60‑09‑3), hydrochloric acid (37%, CAS: 7647‑01‑0), sodium nitrite (CAS: 7632‑00‑0), acetylacetone (≥ 99%, Cat. No. 109205), hydrazine hydrate (≥ 98%, CAS: 7803‑57‑8), semicarbazide hydrochloride (CAS: 3067‑33‑0), thiosemicarbazide (≥ 99%, Cat. No. T1277), ethanol (CAS: 64‑17‑5), petroleum ether 60–80 °C (≥ 95%, CAS: 8032‑32‑4), benzene (≥ 99%, Cat. No. 270709), and acetone (≥ 99%, Cat. No. 34850). All solvents were of analytical grade and were dried according to standard laboratory procedures prior to use. No further purification was required unless otherwise stated. Solvents were dried according to the Laboratory Chemicals Purification Handbook (ethanol over anhydrous Mg followed by distillation, pp. 25–26; acetone over CaH₂ followed by distillation, pp. 40–41; benzene over Na followed by distillation, pp. 55–56; and petroleum ether over benzophenone followed by distillation, pp. 52–53). Commercially available scoured, woven polyester (PET) fabric with weight of (165 g/m^2^) provided from ElMahalla Elkobra Co., Egypt. Sera Sperse M-IS (anionic dispersing agent) was supplied from DyStar. Sodium hydrosulfite and sodium hydroxide pellets were bought from local market.

### Methods

The performed reactions to obtain the pyrazole derivatives were done using three different strategies (conventional, grinding and microwave irradiation). Microwave reactions were carried in Anton Paar monowave 300 with using “10 mL” borosilicate glass vials while the grinding was done in a porcelain mortar with pestle. The same amounts of reactants were used in the three reaction methods. In microwave and grinding techniques, the reactions proceeded without solvent. Thin layer chromatography (TLC) was used as a guiding tool for the reaction’s accomplishments and for controlling the homogeneity of the newly prepared derivatives. The formed solid product was washed with methanol three times then recrystallized from an appropriate solvent.

#### Synthesis of 3-(2-(4-(phenyldiazenyl)phenyl)hydrazono)pentane-2,4-dione (1)

A solution of p-amino azobenzene (0.01 mol, 1.79 g) in H_2_O (5 mL) and conc. HCl (5 mL) was cooled at 0–5 °C. Subsequently, a solution of sodium nitrite (0.01 mol, 0.68 g) was inserted with stirring. The resulting diazonium salt was added to a cold solution of mixture of CH_3_COONa (0.05 mol, 4 g) and acetyl acetone (0.01 mol, 1 mL) in ethanol (20 mL) for 2 h. The reaction progression was monitored using TLC where the eluent was ethanol/petroleum ether (60:80) (7:3). After that, the product was recrystallized from methanol to produce compound **1**.

(m.p 210–212 °C). IR (cm^− 1^) ʋ: 3167 (NH), 1676 (C = O), 1648 (C = N), 1589 (N = N). ^1^H-NMR (300 MHz, DMSO- d_6_) δ(ppm): 2.30 (s, 6 H, 2CH_3_), 7.43–7.84 (m, 9 H, Ar-H), 11.95 (s, 1H, NH, D_2_O exchangeable). ^13^C-NMR (300 MHz, DMSO-d_6_) δ (ppm): 25.4, 111.93, 117.2, 117.4, 122.9, 124.7, 129.9, 131.6, 131.9, 143.9, 145.4, 149.3, 152.5, 160.6 and 161.3. MS (m/z): 308 (M^+^, 10.21%). Anal. Calcd for C_17_H_16_N_4_O_2_ (308): C, 66.23; H, 5.19; N, 18.18. Found: C, 66.01; H, 5.37; N, 18.22.

#### General preparation method of compounds (2–4)

A solution of compound **1** (0.01 mol, 3.08 g) in ethanol (25 mL) with each of hydrazine hydrate (0.01 mol, 0.5 mL), semicarbazide hydrochloride (0.01 mol, 1.11 g) and thiosemicarbazide (0.01 mol, 0.91 g) was refluxed for 2–6 h. The reactions progress was monitored via TLC using acetone/benzene (6:4) as an eluent. The obtained precipitate after cooling was filtered off, washed with ethanol (100 mL) and recrystallized from the suitable solvent to give compounds **2–4**, respectively.

### 3,5-Dimethyl-4-((4-(phenyldiazenyl)phenyl)diazenyl)-1 H-pyrazole (2)

(m.p 252–254 °C). IR (cm^− 1^) ʋ: 3192 (NH), 1648 (C = N), 1554 (N = N). ^1^H-NMR (300 MHz, DMSO-d_6_) δ (ppm): 2.30 (s, 6 H, 2CH_3_), 7.51–7.93 (m, 9 H, Ar-H), 8.60 (s, 1H, NH, D_2_O exchangeable). ^13^C-NMR (300 MHz, DMSO-d_6_) δ (ppm): 20.2, 113.9, 116.0, 117.2, 117.3, 122.8, 122.9, 124.7, 129.9, 131.6, 131.8, 143.9, 145.2, 152.5, 160.7 and 161.2. MS (m/z): 304 (M^+^, 14.26%). Anal. Calcd for C_17_H_16_N_6_ (304): C, 67.11; H, 5.26; N, 27.63. Found: C, 67.32; H, 5.09; N, 27.59.

### 3,5-Dimethyl-4-((4-(phenyldiazenyl)phenyl)diazenyl)-1 H-pyrazole-1-carboxamide (3)

(m.*p* > 300 °C). IR (cm^− 1^) ʋ: 3332, 3235 (NH_2_), 1679 (C = O), 1600 (C = N), 1531 (N = N). ^1^H-NMR (300 MHz, DMSO-d_6_) δ (ppm): 2.30 (s, 6 H, 2CH_3_), 7.55–7.99 (m, 11 H, Ar-H & NH_2_, D_2_O exchangeable). ^13^C-NMR (300 MHz, DMSO-d_6_) δ (ppm): 20.1, 105.4, 112.6, 117.3, 117.4, 122.8, 122.9, 124.7, 129.9, 131.7, 146.1, 149.0, 152.4, 152.5, 160.7, 161.5 and 181.6. MS (m/z): 347 (M^+^, 8.72%). Anal. Calcd for C_18_H_17_N_7_O (347): C, 62.25; H, 4.90; N, 28.24. Found: C, 62.41; H, 4.88; N, 28.09.

### 3,5-Dimethyl-4-((4-(phenyldiazenyl)phenyl)diazenyl)-1 H-pyrazole-1-carbothioamide (4)

(m.*p* > 300 °C). IR (cm^− 1^) ʋ: 3321, 3206 (NH_2_), 1648 (C = N), 1595 (N = N), 1244 (C = S). ^1^H-NMR (300 MHz, DMSO-d_6_) δ (ppm): 2.30 (s, 6 H, 2CH_3_), 5.95 (s, 2 H, NH_2_, D_2_O exchangeable), 7.54–7.96 (m, 9 H, Ar-H). ^13^C-NMR (300 MHz, DMSO-d_6_) δ (ppm): 20.8, 105.8, 111.8, 113.9, 116.0, 117.2, 117.3, 122.2, 122.8, 122.9, 129.9, 131.7, 131.8, 145.2, 149.1, 152.5 and 201.2. MS (m/z): 363 (M^+^, 2.85%). Anal. Calcd for C_18_H_17_N_7_S (363): C, 59.50; H, 4.68; N, 27.00; S, 8.82. Found: C, 59.39; H, 4.78; N, 26.98; S, 8.85.

### Characterization

TLC was performed through precoated plates of silica gel “Merck Kiesel gel 60F_254_, BDH”. Melting points of all newly prepared compounds were measured using digital electric Stuart apparatus (SMP3). Infrared spectra (IR, cm^− 1^) were recorded using KBr disks by PerkinElmer 293 spectrophotometer. ^1^H and^13^ C-NMR spectra were determined on Varian Mercury Spectrometer (300 MHz) in solvent DMSO-d_6_ with an internal standard tetramethyl silane TMS. Multiplicity is symbolized as m “multiple”, q “quartet”, t “triplet”, d “doublet”, s “singlet” or combinations therefrom. Coupling constants (J) were measured in Hz and chemical shift (δ) in ppm. Mass spectra were measured using the electrons ionization technique on a Shimadzu Gas Chromatography (GC-2010, 70 eV) instrument mass spectrometer. Elemental microanalyses were recorded on a PerkinElmer analyzer CHN-2400 and the good agreement microanalyses within ± 0.4% of the theoretical values.

### Microwave and grinding synthesis

The amounts of reactants and synthetic procedures of microwave-aided and grinding methods are like the conventional heating technique, but without solvent. The same amounts of reactants as in the conventional procedure. The reaction vial was irradiated at 115 °C under pressure (2–4 bar) and power (250–450 W) for 1–2 min (Table 1) with 750 rpm magnetic stirring rate. Grinding technique was applied using a porcelain mortar and a pestle for 9–15 min. Applying the three methods, the same reaction’s final product was the same in TLC, m.p., and mixed m.p. in both techniques. The environmental and economic advantages of the grinding and microwave-assisted methods, including shorter reaction times, reduced solvent consumption, and higher product yields compared with the conventional heating procedure, are summarized in Table 1, demonstrating that these green synthetic approaches provide lower energy requirements and improved sustainability.

### Dyeing of polyester fabric

2 g of PET fabric was introduced in cups containing 1, 2 and 3% owf (on weight fabric) of dyes (**1–4**) with a 1:50 liquor ratio and 1, 2 and 3% owf (on weight fabric) anionic dispersing agent (Sera Sperse MIS), respectively. The dyeing was performed at 120 °C and pH 4 for 60 min using INFRA COLOUR dyeing machine, R.B. Electronic & Engineering Private Limited, India. The dyed fabric was rinsed in warm water and treated in a solution containing sodium dithionite (2 g/L) and sodium hydroxide (2 g/L) (reduction clearance step) at 60 °C for 10 min and a liquor ratio of 1:40. Subsequently, the fabric was thoroughly rinsed in cold water, neutralized with acetic acid (1 g/L) at 40 °C for 5 min followed by rinsing in tap water and drying at an ambient temperature^53^.

### Measurements and analyses

#### UV-visible spectroscopy

A dilute solution of disperse diazo dyes (5 × 10^− 5^ M) was prepared in DMF/distilled water (1:1) and the absorption spectra of the dye solutions was recorded by a Shimadzu UV2401PC UV–Visible spectrophotometer. The spectrophotometer was calibrated with a blank solution of DMF/distilled water (1:1) before measurements. Absorbance readings of the dye solutions were taken over the range of 350–700 nm.

#### Color strength and CIELAB

The relative color strength (K/S) of the dyed PET fabrics was recorded using a Hunter Lab UltraScan Pro spectrophotometer (USA) with D65 illuminant, 10° standard observer. The K/S values were calculated from Kubelka Munk Eq. (1)^54^.

4 $$\:\mathrm{K}/\mathrm{S}=\frac{(1-\mathrm{R})}{2\mathrm{R}}-\frac{(1-\mathrm{R}\circ\:)}{2\mathrm{R}\circ\:}$$

Where: K: Absorption coefficient, S: Scattering coefficient, R, R°: Decimal fraction of the reflectance of the dyed and undyed fabrics, respectively.

The colorimetric data of whole dyed samples were expressed in the CIELAB color space including L*, a*, b*, C*, h coordinates. L* (lightness) represents the brightness of the color with a scale from 0 to 100 (black-white, respectively, a* represents the red-green axis in which the positive value indicates redness and the negative value point greenness, b* represents the yellow-blue axis where the positive value refers to yellowness and the negative value denotes to blueness, C* is a measure of the color saturation and h (hue) is used to determine the color position on 360° spectrum (0°:red, 90°: yellow, 180°:green, 360°: blue). Also, the difference between two colors (ΔE) is measured using Eq. (2)^55^.

5 $$\:{\Delta\:}\mathrm{E}=[{({\varDelta\:{\mathrm{L}}^{\mathrm{*}})}^{2}+({\varDelta\:{\mathrm{a}}^{\mathrm{*}})}^{2}+\left({\varDelta\:{\mathrm{b}}^{\mathrm{*}})}^{2}\right]}^{0.5}$$

Where, ΔE = the difference between the dyed fabric’s color and the standard, L* = the lightness from 0 (black) − 100 (white), a* = red (+ ve) - green (-ve) ratio, b* = yellow (+ ve) – blue (-ve) ratio.

#### Fastness properties

##### Color fastness to washing

Color fastness to washing was evaluated according to the test method ISO 105-C06:2010. The dyed fabrics were stitched to a white piece of cotton, wool and PET fabrics then immersed into an aqueous solution containing (5 g/L) nonionic detergent (liquor ratio 1:50) at 50 °C for 30 min. Fabrics were rinsed twice with occasional hand squeezing and dried. After that, the grey scale was used for assessing the color change and staining^56^.

##### Color fastness to rubbing

Color fastness to rubbing was examined in accordance with ISO 105-X12:2016 using crock meter. The test is designed to determine the resistance of the color of the dyed fabric to satin other materials or surfaces by rubbing. The test can be carried out using a dry rubbing cloth (dry rubbing) and a wet rubbing cloth (wet rubbing). The change in color and the staining of the adjacent fabrics was recorded using the grey scale^57^.

##### Color fastness to perspiration

Color fastness to perspiration is a measurement of the ability of the dyed fabric to retain its color under the effect of human perspiration. Two artificial perspiration solutions were prepared according to the standard method ISO 105-E04:2013. The acidic perspiration solution contains (0.5 g/L) L-histidine monohydrochloride monohydrate, (10 g/L) sodium chloride and (1 g/L) sodium dihydrogen orthophosphate dihydrate. The alkaline perspiration solution was prepared using (0.5 g/L) L-histidine monohydrochloride monohydrate, (10 g/L) sodium chloride and (1 g/L) disodium hydrogen orthophosphate dihydrate. Then the pH was adjusted to 5 using acetic acid and 8 using sodium hydroxide for the acidic and alkaline solutions, respectively. The color change and staining were defined by reference to the grey scale^58^.

##### Color fastness to light

Color fastness to light was carried out in accordance with the method ISO 105-B02:2014 using carbon arc lamp with continuous light for 35 h. The effect on the color of dyed fabrics was measured using the blue scale for color change and staining^59^.

#### Antimicrobial activity

Antimicrobial activity of the dyed specimens was studied toward different strains. The investigation included microorganisms such as *S. aureus* (gram positive, ATCC 6538) and *E. coli* (gram negative, ATCC 25922) bacteria in addition to *C. albicans* (fungi, ATCC 10231).

The inoculation of microorganisms was prepared from fresh broth cultures using nutrient broth medium that were incubated overnight at 37 °C, incubation shaking speed at 150 rpm. The inoculum suspension of each strain was prepared and adjusted to approximately 0.5 McFarland standard (1.5 × 10^8^/mL). 15µL of both bacterial and fungal suspensions were separately inoculated with 25 mL of the sterile nutrient broth medium. Antimicrobial effectiveness expressed by (%) reduction of bacterial count i.e. by calculating colony forming unit (CFU) of the tested strains in absence and presence of the dyed fabrics after incubation at 37 °C for 24 h, incubation shaking speed at 150 rpm. All results were expressed according to Eq. (3)^60^.

6 $$\:\mathrm{R}\mathrm{e}\mathrm{l}\mathrm{a}\mathrm{t}\mathrm{i}\mathrm{v}\mathrm{e}\:\mathrm{R}\mathrm{e}\mathrm{d}\mathrm{u}\mathrm{c}\mathrm{t}\mathrm{i}\mathrm{o}\mathrm{n}\:\left({\%}\right)\:=\frac{\mathrm{A}-\mathrm{B}}{\mathrm{A}}\times\:100$$

where, A and B represent the number of microorganisms grown in the absence and presence of the dyed fabrics, respectively.

#### Ultraviolet protection factor (UPF)

Ultraviolet protection factor (UPF) is a measurement of the efficiency of textiles to absorb or block UV radiation. UPF of the dyed fabrics was recorded in accordance with the Australian/New Zealand standard method (AS/NZS 4399:1996)^61^ using UV/Visible double-beam Spectrophotometer (JASCO V-750) from 280 to 400 nm at an interval of 5 nm. Equation (4) is used to estimate the UPF value as follows:

7 $$\:\mathbf{U}\mathbf{P}\mathbf{F}=\frac{{\sum\:}_{290}^{400}\:{\boldsymbol{E}}_{\boldsymbol{\lambda\:}}\:\times\:\:\:{\boldsymbol{S}}_{\boldsymbol{\lambda\:}}\:\times\:\:\varDelta\:\:\boldsymbol{\uplambda\:}}{{\sum\:}_{290}^{400}\:{\boldsymbol{E}}_{\boldsymbol{\lambda\:}\:}\times\:\:{\boldsymbol{S}}_{\boldsymbol{\lambda\:}}\:\times\:\:{\boldsymbol{T}}_{\boldsymbol{\lambda\:}\:}\times\:\:\varDelta\:\:\boldsymbol{\uplambda\:}\:\:\:\:}$$

where, *E*_λ_ = relative erythemal spectral effectiveness, *S*_λ_ = solar spectral irradiance (W.M^− 2^.nm^− 1^), *T*_λ_ = spectral transmittance of the fabric, Δλ = the wavelength step (nm), λ = the wavelength (nm).

## Results and discussion

### Synthesis and characterization of the disperse azo dyes

**Fig. 1:**
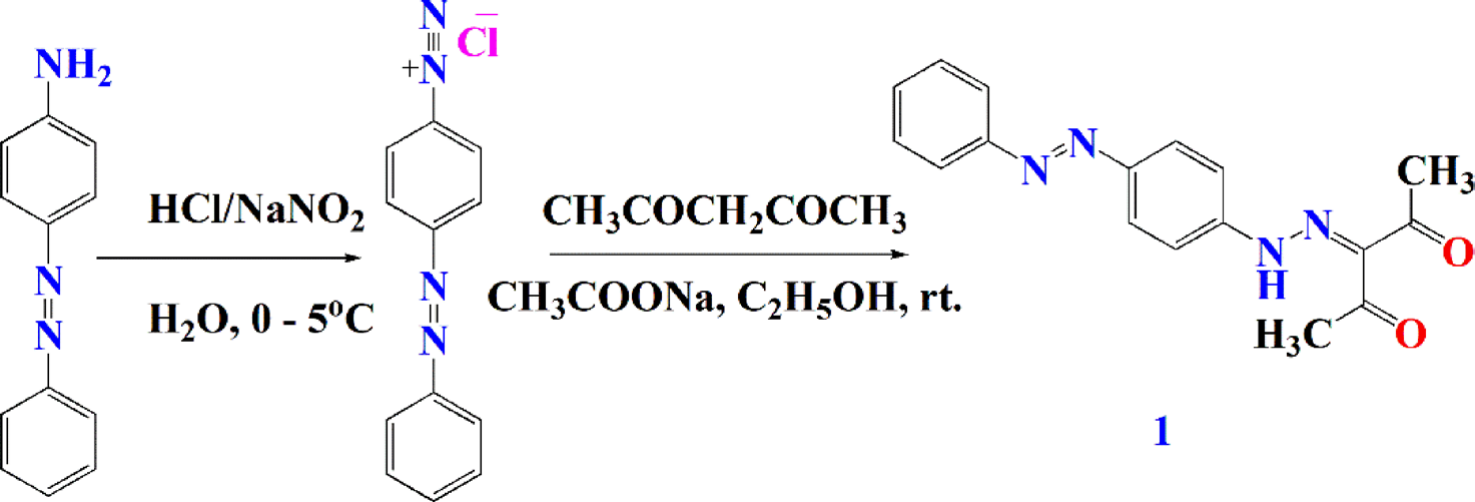
Preparation of dye **1**.

Diazonium salts are significantly regarded as electrophilic reagents especially when coupled with various active methylene compounds e.g. acetylacetone, malononitrile and diethyl malonate in presence of few drops of alkali This coupling reaction often leads to the formation of a wide range of colored azo compounds^62^. Considering this confirmed scientific fact, p-aminoazobenzene has been diazotized with sodium nitrite in the presence of conc. HCl to give the diazonium salt (electrophilic reagent) (Fig. 1). So, the obtained diazonium salt was coupled with active methylene derivative entities acetylacetone in ethanol at 20–25 °C to yield the corresponding azo analog **1**. Compound **1** was utilized as an intermediate for the synthesis of the target 3,5-dimethyl pyrazole derivatives **2–4**. The construction precursor was illustrated depending on its elemental analysis and spectral data. IR spectrum of compound **1** was approved by the appearance of C = O band at 1676 cm^− 1^. Its^1^H-NMR spectrum further approved the desired structure as it showed one singlet peak of the two methyl moieties at 2.30 ppm. Its molecular ion peak at m/z 308 [M^+^] in mass spectrum corresponding to molecular formula C_17_H_16_N_4_O_2_.

**Fig. 2:**
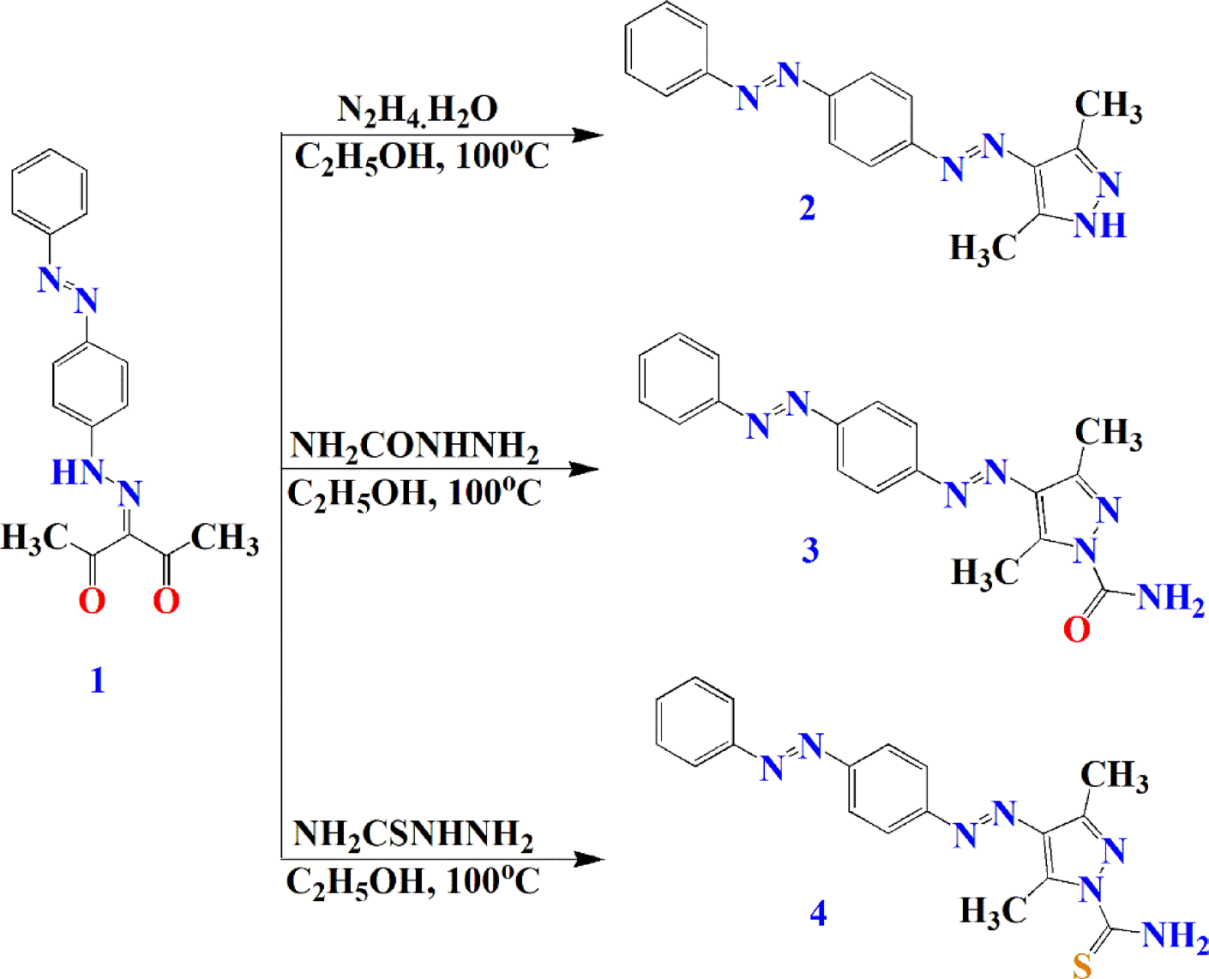
Preparation of dyes **2–4**.

Dyes **2–4** were obtained through condensation of the diketo derivative **1** with hydrazine hydrate, semicarbazide and thiosemicarbazide, respectively (Fig. 2).

**Fig. 3:**
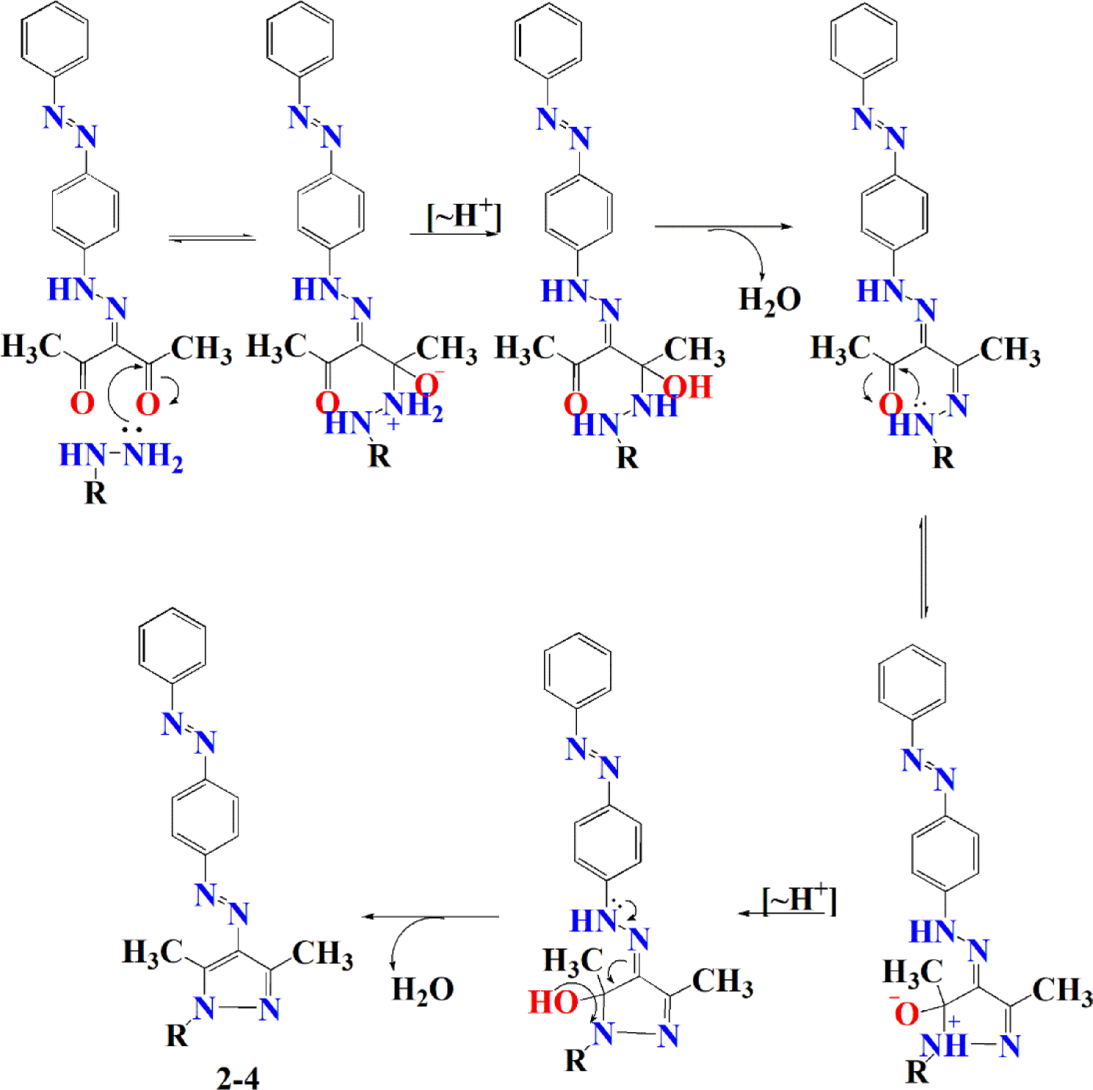
The supposed mechanism for preparation of dyes **2–4**.

The formation of the condensed products (**2–4**) may occur first through the nucleophilic attack of NH_2_ of the hydrazine derivative on one of the carbonyl groups of the di-carbonyl compound **1**, then 1,3-proton shift from nitrogen to oxygen in the formed intermediate hydrazinium cation followed by loss of one molecule of water. Similarly, the second NH-R group functionality undergoes the same nucleophilic condensation reaction followed by cyclization with the second C = O group to give the 3,5-dimethyl pyrazole derivatives **2–4**. The supposed mechanism for the keto derivative **1** transformation into the corresponding 3,5-dimethyl pyrazole **2–4** was given in (Fig. 3).

Spectral data of the prepared 3,5-dimethyl pyrazoles **2–4** illustrated their expected structures. The characteristic absorption lack for carbonyl group of ester derivative **1** and the presence of amidic carbonyl appeared at the range of 1676 cm^− 1^ in IR chart evidenced complete diazonium salts consuming and formation of the targeted compounds. IR spectra of compounds **2–4** were devoid of νC= O of ketonic and showing an amidic carbonyl at 1679 cm^− 1^ from compound **3** and νC = S at 1244 cm^− 1^ from compound **4** which support the proposed structures.

### Comparison between conventional, grinding, and microwave techniques

Green chemistry metrics describe aspects of a chemical process relating to the principles of green chemistry. The metrics serve to quantify the efficiency or environmental performance of chemical processes and allow changes in performance to be measured.

The most favorable approach for a definite reaction was determined by calculating the yield economy (YE). Yield economy can be calculated using Eq. (5):

1 $$\:YE=\:\frac{yield{\%}}{Reaction\:time\:"min"}$$

Moreover, other parameters utilized for the differentiation and comparison between the three methods are reaction mass efficiency (RME) and optimum efficiency (OE). Both parameters are calculated from Eqs. (6 and 7), respectively.

2 $$\:\mathrm{R}\mathrm{M}\mathrm{E}\:=\frac{Weight\:of\:isolated\:product\:}{Weight\:of\:reactants}$$

3 $$\:OE=\:\frac{RME}{AE}\:x\:100$$

Where, “AE” refers to the atomic economy.

To determine the most suitable strategy to obtain the desired compounds, various parameters including reaction times, yields, and green chemistry metrics (YE, AE, RME, and OE) were measured and calculated for the three methods, as reported in )Table 2(. The isolated products obtained using conventional, grinding, and microwave techniques were identical in m.p., mixed m.p., and TLC. Although the same amounts of reactants were used, noticeable differences were observed in the reaction times and yields for each strategy. Microwave-assisted reactions provided the highest yields in the shortest reaction times, followed by grinding and then conventional methods. YE, RME, and OE values were higher for the microwave and grinding methods compared with the conventional method, while AE remained identical across all techniques. the microwave-assisted method offers greener conditions due to shorter reaction times, reduced or no solvent usage, and higher product yields. Consequently, microwave irradiation can be considered a more favorable and environmentally friendly technique compared with grinding and conventional heating.

For compound 2, the microwave metrics were calculated as follows:

Yield Economy (YE) = $\:\frac{{{\mathrm{yield}}\% \:}}{{{\mathrm{time}}\:''{\mathrm{min''}}}}$= $\:\frac{95}{1}$ = 95.

Atom Economy (AE) = $\:\frac{\mathrm{m}\mathrm{o}\mathrm{l}\mathrm{e}\mathrm{c}\mathrm{u}\mathrm{l}\mathrm{a}\mathrm{r}\:\mathrm{w}\mathrm{e}\mathrm{i}\mathrm{g}\mathrm{h}\mathrm{t}\:\mathrm{o}\mathrm{f}\:\mathrm{p}\mathrm{r}\mathrm{o}\mathrm{d}\mathrm{u}\mathrm{c}\mathrm{t}}{\mathrm{s}\mathrm{u}\mathrm{m}\:\mathrm{o}\mathrm{f}\:\mathrm{m}\mathrm{o}\mathrm{l}\mathrm{e}\mathrm{c}\mathrm{u}\mathrm{l}\mathrm{a}\mathrm{r}\:\mathrm{w}\mathrm{e}\mathrm{i}\mathrm{g}\mathrm{h}\mathrm{t}\mathrm{s}\:\mathrm{o}\mathrm{f}\:\mathrm{r}\mathrm{e}\mathrm{a}\mathrm{c}\mathrm{t}\mathrm{a}\mathrm{n}\mathrm{t}\mathrm{s}}$ × 100 = $\:\frac{304}{308+50}$ × 100= 84.92%.

Reaction Mass Efficiency (RME) = $\:\frac{\mathrm{m}\mathrm{a}\mathrm{s}\mathrm{s}\:\mathrm{o}\mathrm{f}\:\mathrm{p}\mathrm{r}\mathrm{o}\mathrm{d}\mathrm{u}\mathrm{c}\mathrm{t}\:}{\mathrm{t}\mathrm{o}\mathrm{t}\mathrm{a}\mathrm{l}\:\mathrm{m}\mathrm{a}\mathrm{s}\mathrm{s}\:\mathrm{o}\mathrm{f}\:\mathrm{r}\mathrm{e}\mathrm{a}\mathrm{c}\mathrm{t}\mathrm{a}\mathrm{n}\mathrm{t}\mathrm{s}}$ × 100 = $\:\frac{2.888}{3.08+.50}$ = 80.67%.

Optimum Efficiency (OE) = $\:\frac{\mathrm{R}\mathrm{M}\mathrm{E}\:}{AE}$ × 100 = $\:\frac{84.92\:}{80.67}$ × 100 = 94.99%.

Similar calculations were performed for the other compounds **2–4** and summarized in Table 2.

**Table 1:** Comparative study between conventional, grinding and microwave techniques (Time and Yield)*.

Dye	Time (min)			Yield (%)		
	C.	G.	M.	C.	G.	M.
2	120	9	1	58	83	95
3	360	12	2	61	82	94
4	360	15	2	60	84	91

*C. → conventional, G. → grinding and M. → microwave.

**Table 2:** Comparative study between conventional, grinding and microwave techniques (YE, RME, OE and AE)*.

Dye	YE			RME			OE			AE
	C.	G.	M.	C.	G.	M.	C.	G.	M.	
2	0.4833	9.22	95	43.64	70.48	80.67	51.39	83.00	94.99	84.92
3	0.1694	6.83	47	45.52	67.91	77.85	54.96	82.00	94.00	82.82
4	0.1667	5.6	45.5	48.94	76.42	82.79	53.79	84.00	91.00	90.98

*C. → conventional, G. → grinding and M. → microwave.

### Dyeing of PET fabrics

**Fig. 4:**
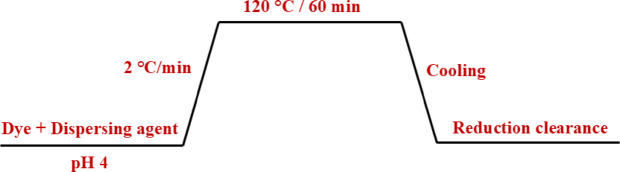
Chart of dyeing procedure of PET using exhaustion dyeing method.

The dyeing process of polyester fabrics with the newly synthesized disperse diazo dyes is demonstrated in Fig. 4. Polyester fabrics were dyed with three shades (1, 2 and 3% owf) using exhaustion dyeing technique (high temperature-pressure approach). After that, the dyed samples were treated with sodium hydrosulfite and sodium hydroxide (reduction clearance step) to remove unfixed dyes and auxiliaries from the fabric surface and ensure optimal fastness and shade.

### UV-visible spectroscopy

**Fig. 5:**
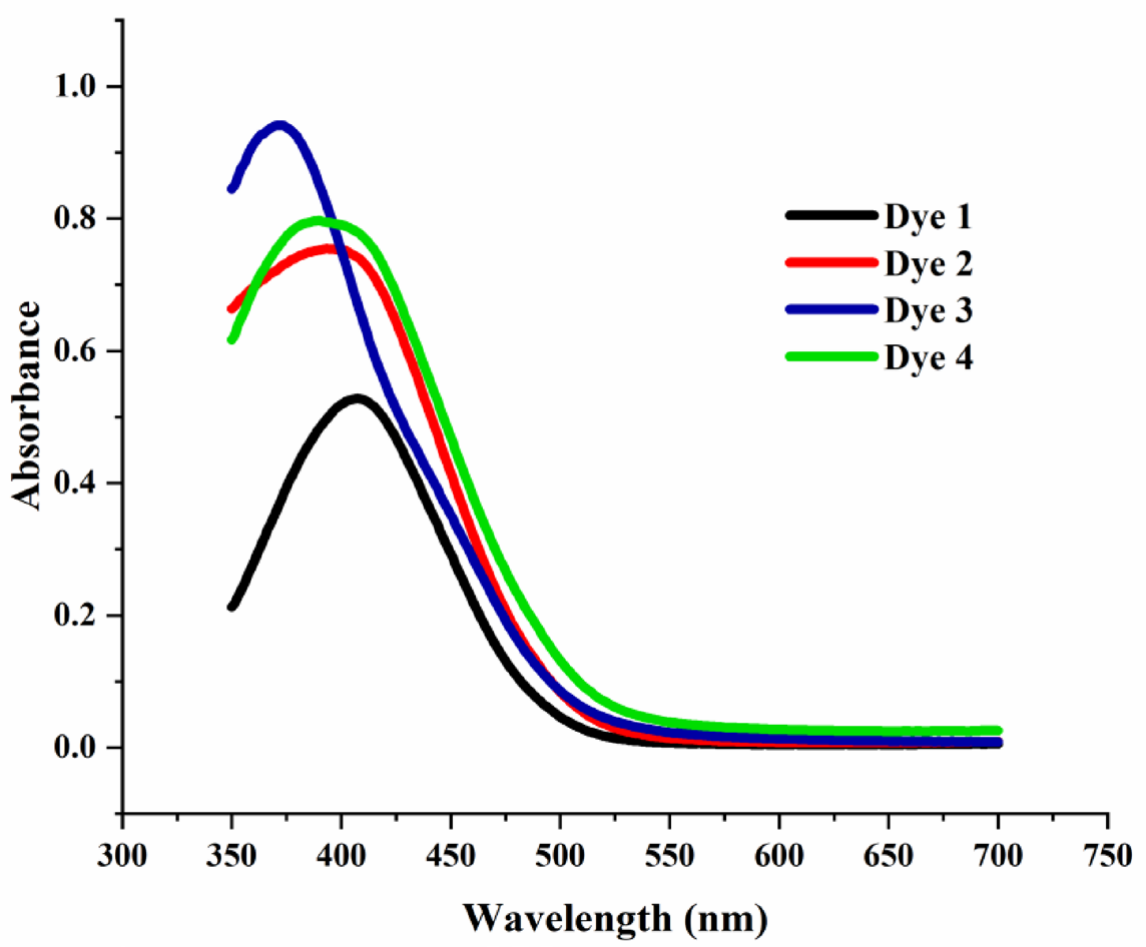
UV-visible spectra of the synthesized disperse diazo dyes.

The UV–visible absorption spectra of the synthesized dyes showed λmax values in the range of 370–410 nm as shown in Fig. 5. The observed differences can be attributed to variations in conjugation and the electronic effects of substituents on the pyrazole ring, without making speculative mechanistic claims about isomerization or hydrogen shifts under UV irradiation.

**Fig. 6:**
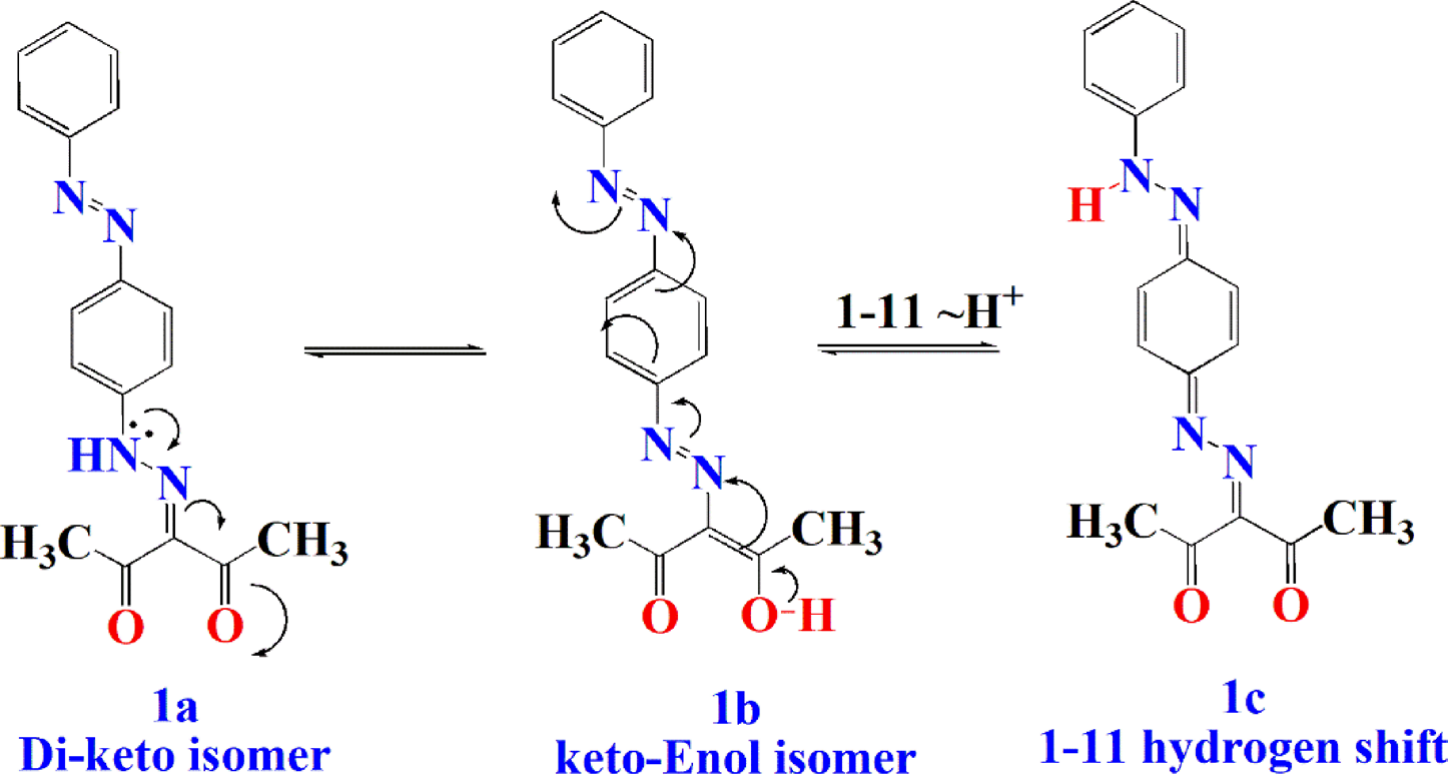
Conjugation occurred in dye 1 under UV irradiation.

These bands were produced by the conjugated systems formed in each dye e.g. dye **1** is found in two isomer forms as di-keto isomer **1a** and keto-enol isomer **1b**. Both isomers are formed in solution and in the presence of UV irradiation, the longest conjugation occurred in one system between O_1_ and N_11_ forming 1–11 hydrogen shift to give isomer **1c** with wavelength 408 nm (Fig. 6).

**Fig. 7:**
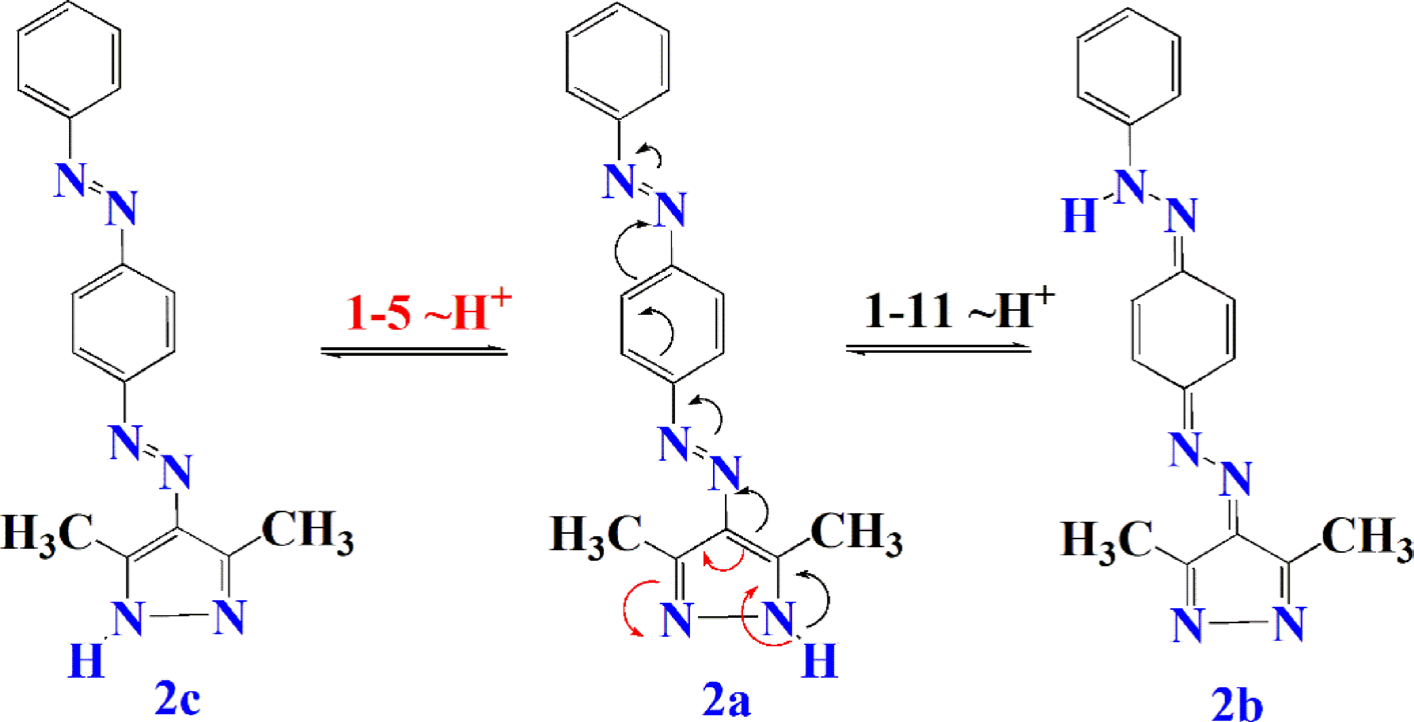
Conjugation occurred in dye 2 under UV irradiation.

In case of dye **2**, The presence of two differently oriented conjugation pathways in isomers **2b** and **2c** leads to reduced effective π-electron delocalization across the molecule. This partial disruption of conjugation results in a hypsochromic shift, giving a lower absorption wavelength 394 nm compared to dye 1 (Fig. 7).

**Fig. 8:**
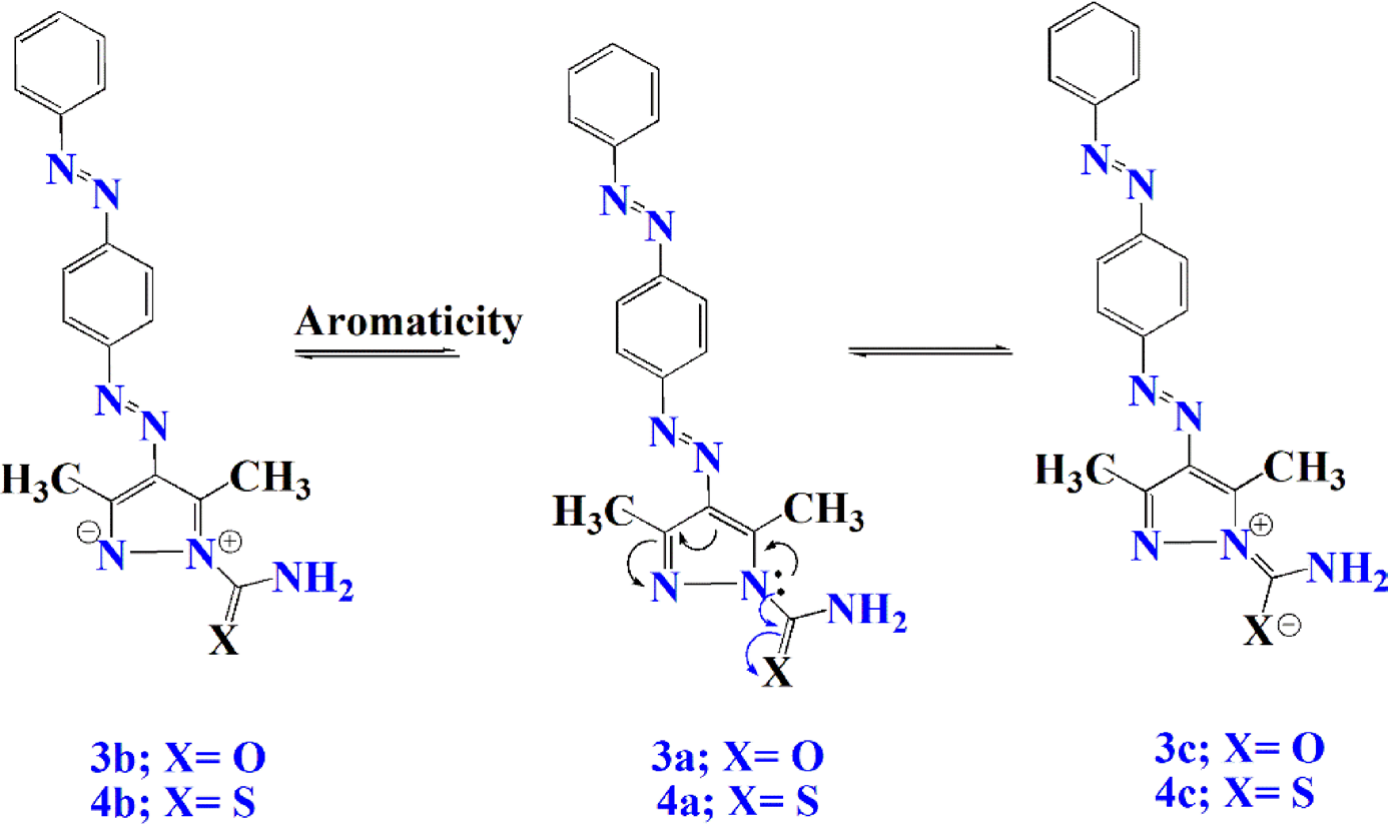
Conjugation occurred in dyes 3 and 4 under UV irradiation.

In dyes **3** and **4**, the most stable isomers are **3a** and **4a**. The existence of amide and thioamide groups reduce the conjugation systems forming aromaticity to produce isomers **3b** and **4b**. Another conjugation system takes place with the outer groups which give isomers **3c** and **4c**. Consequently, the absorption wavelength of both dyes decreased to 373 and 390 nm, respectively. The slightly higher λ_max_ of dye **4** compared to dye **3** may be attributed to the higher polarizability of sulfur compared with oxygen, which enhances electron delocalization within the chromophore system. (Fig. 8).

The variation in λ_max_ values among the dyes can also be attributed to the electronic effects of the substituents attached to the pyrazole ring. Electron-withdrawing groups such as amide and thioamide modify the electron density and conjugation pathway within the chromophoric system, which consequently affects the absorption maxima.

### Color strength and CIELAB

Color strength (K/S) and colorimetric data of PET fabrics dyed using the four synthesized pyrazole dyes **1–4** at all three concentrations (1%, 2% and 3% owf) were measured. The obtained results are given in Tables 3 and 4, from which it is clear that, increasing the dye concentration of the prepared dyes accordingly increases the color strength of the dyed samples with a higher depth of shade as 1%, 2% and 3% respectively, exhibited better dye build-up and fixation possibly because of the higher fixation efficiency of dye **4**,** 3**,** 2** and **1**. Moreover, the average color differences ΔE (calculated from the CIE L*a*b* coordinates) of the dyed fabrics exhibited a very good levelling properties.

### Fastness properties

The fastness properties to washing as well as acid and alkaline perspiration of the dyed PET samples using the prepared dyes 1-4 at 1%, 2% and 3% owf dye concentration, liquor ratio 1:50, pH 4 and 120°C and through the dyeing times of 60 min are given in Table 5. The fastness to wash and perspiration of the dyed fabrics was very good with slightly better fastness at 1% dye concentration for all dyes 1-4 as the low dye concentration resulting in better dye fixation. 

The color fastness to rubbing and light of the dyed specimens using dyes 1-4 is outlined in Table 6. The dyed PET fabrics at different dye concentrations showed very good to good ratings to rubbing fastness with slightly lower rating at high dye concentration 3% due to the presence of some unfixed dye molecules on the surface of fabrics. While the light fastness of the samples dyed with the synthesized dyes 1-4 displayed approximately similar values with very good ratings. All the synthesized dyes 1-4 showing similar fastness properties probably because of having the same pyrazole chromophoric system.

**Table 3:**
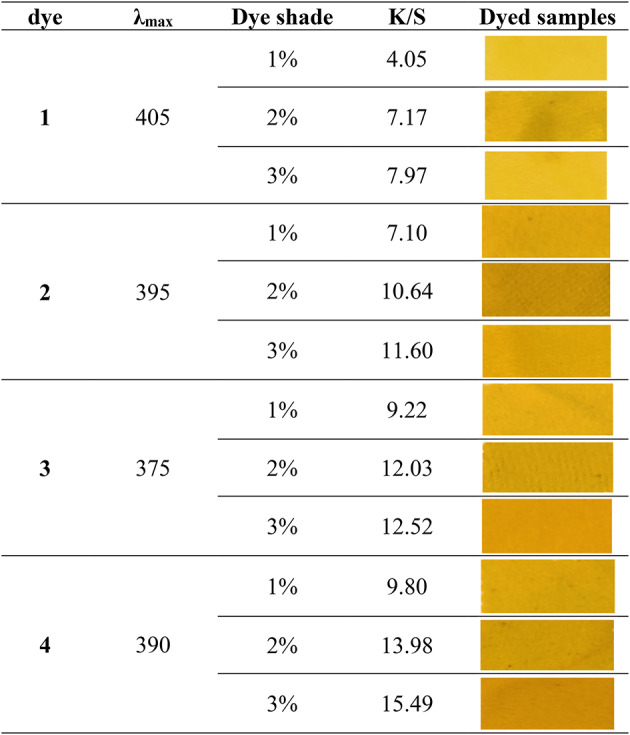
Color strength (K/S) of dyed PET fabrics using the synthesized pyrazole dyes 1–4.

**Table 4:** colorimetric data of dyed PET fabrics using the synthesized pyrazole dyes **1-4**.

Dye	Dye shade	L*	a*	b*	c*	h	dE
1	1%	77.71	-2.94	45.42	45.51	93.70	54.92
	2%	71.63	2.71	55.55	55.62	87.20	65.56
	3%	74.93	-1.06	45.86	45.88	91.33	55.51
2	1%	73.08	4.63	56.88	57.07	85.35	66.59
	2%	67.16	7.67	60.06	60.55	82.72	70.93
	3%	71.21	7.80	69.28	69.72	83.58	79.27
3	1%	74.02	1.99	51.11	51.15	87.77	60.72
	2%	69.94	4.84	57.48	57.69	85.19	67.70
	3%	69.83	12.91	68.57	69.77	79.34	79.28
4	1%	72.77	4.42	59.58	59.74	85.76	69.30
	2%	70.28	6.53	63.33	63.66	84.11	73.47
	3%	68.29	10.47	67.39	68.20	81.17	78.10

**Table 5:** Washing and perspiration fastness properties of dyed PET fabrics using the synthesized pyrazole dyes 1-4.

Dye	Dye shade	Washing fastness				Perspiration fastness							
						Acidic				Alkaline			
		St.	St.*	St.**	Alt.	St.	St.*	St.**	Alt.	St.	St.*	St.**	Alt.
1	1%	4-5	4-5	4-5	4	4-5	4-5	4-5	4	4-5	4-5	4-5	4-5
	2%	4	4	4	4	4	4	4	4	4	4	4	4
	3%	4	4	4	4	4	4	4	4	4	4	4	4
2	1%	4-5	4-5	4-5	4	4-5	4-5	4-5	4	4-5	4-5	4-5	4-5
	2%	4	4	4	4	4	4	4	4	4	4	4	4
	3%	4	4	4	4	4	4	4	4	4	4	4	4
3	1%	4-5	4-5	4-5	4	4-5	4-5	4-5	4	4-5	4-5	4-5	4-5
	2%	4	4	4	4	4	4	4	4	4	4	4	4
	3%	4	4	4	4	4	4	4	4	4	4	4	4
4	1%	4-5	4-5	4-5	4-5	4-5	4-5	4-5	4	4-5	4-5	4-5	4-5
	2%	4	4	4	4	4	4	4	4	4	4	4	4
	3%	3-4	3	3-4	3-4	4	4	4	4	4	4	4	4

St.: staining on cotton; St.*: staining on wool; St.**: staining on polyester: Alt.: alteration

**Table 6:** Rubbing and light fastness properties of dyed PET fabrics using the synthesized pyrazole dyes 1-4.

Dye	Dye shade	Rubbing fastness		Light fastness
		Dry	Wet	
1	1%	4-5	3-4	4-5
	2%	3-4	3-4	4-5
	3%	3	3	5
2	1%	4	4	4-5
	2%	3-4	3-4	5
	3%	3-4	2-3	6
3	1%	4	3-4	5-6
	2%	3-4	3-4	5-6
	3%	3-4	3	5
4	1%	4	3-4	5-6
	2%	3-4	3-4	5-6
	3%	3	2-3	3-4

### Antimicrobial activity

**Fig. 9:**
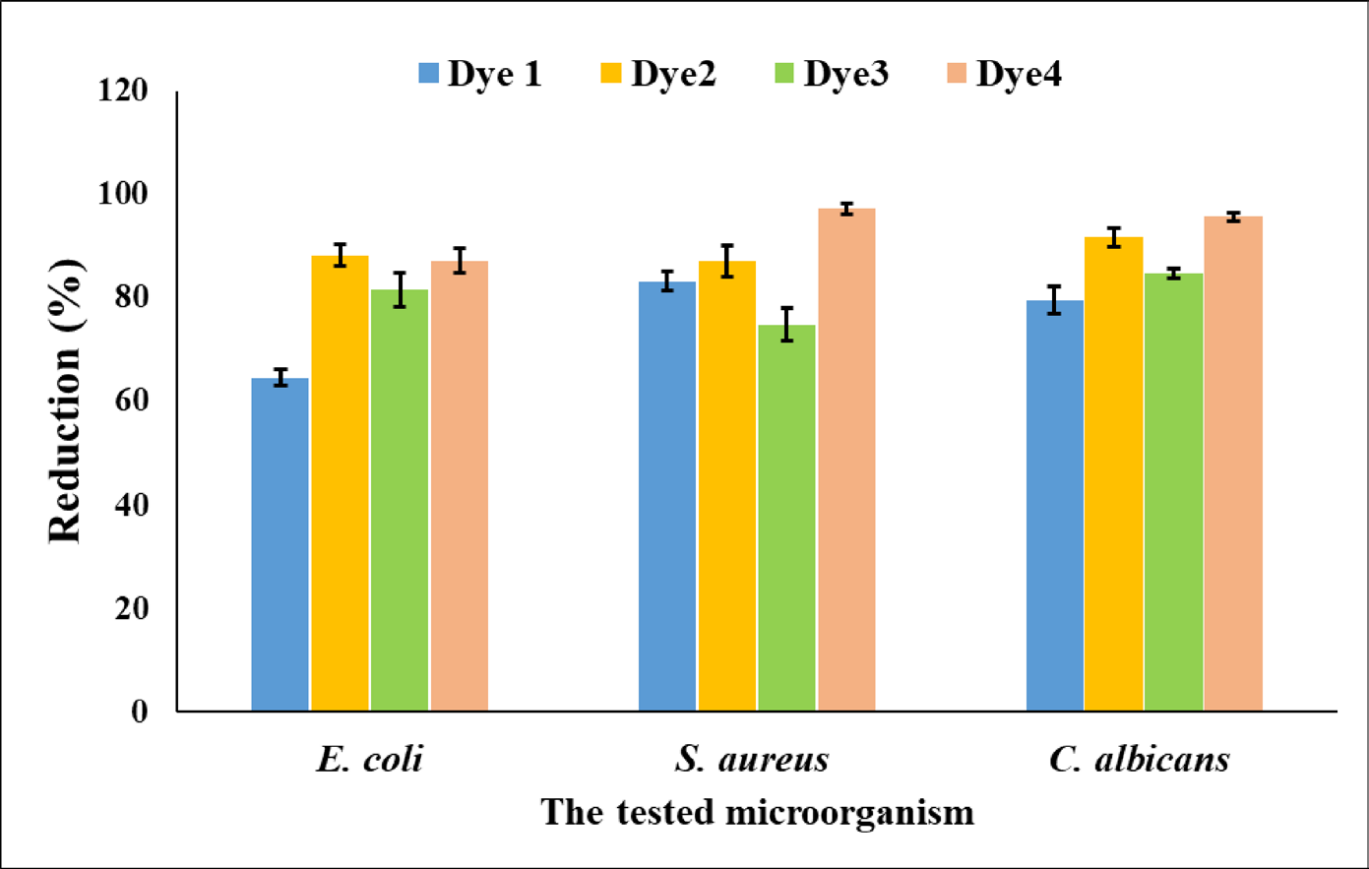
Antimicrobial activity of dyed PET fabrics.

Antimicrobial activity of the dyed PET fabrics using 3% shade was investigated against *S. aureus* (gram +ve bacteria), *E. coli* (gram -ve bacteria) and *C. albicans* (fungi). As seen in Fig. 9, PET fabrics dyed with the synthesized diazo dyes showed excellent antimicrobial activity against the tested microorganisms. It is noteworthy that untreated PET fabrics exhibit negligible antimicrobial activity, as reported in the literature^63^; therefore, the observed activity is mainly attributed to the applied dyes. The observed antibacterial and antifungal activities (up to 97%) may be attributed to the synergistic effect of azo and pyrazole functionalities, as well as the nature of the substituents. These findings are consistent with earlier reports, while demonstrating noticeable improvement in certain performance aspects.^64–66^.

### Ultraviolet protection factor (UPF)

According to Australian/New Zealand standard (AS/NZS 4399:1996), the UV protection categorized to insufficient protection at UPF ˂15, good protection at UPF = 15 to 24, Very good protection at UPF = 25 to 39, Excellent protection at UPF = 40 to 50, 50+. UPF values of undyed and dyed PET fabrics are demonstrated in Table 7. The measured UPF of undyed PET fabric showed that it has inadequate protection against UV radiation. The UPF values of the dyed samples indicated that the PET fabric has gained excellent UV protection after dyeing with the prepared pyrazole azo dyes **1–4**.

**Table 7:** UPF of undyed and dyed PET fabrics using the synthesized pyrazole dyes 1–4.

Dye	Dye shade	UPF
1	1%	34.5
	2%	40.0
	3%	40.5
2	1%	44.3
	2%	46.0
	3%	49.0
3	1%	44.2
	2%	47.0
	3%	51.2
4	1%	44.7
	2%	46.4
	3%	50.2

UPF of undyed PET = 7.1.

## Conclusion

Diazo-scaffold joined pyrazole moieties have been synthesized and developed utilizing several approaches including the conventional method and an environmentally benign and cost-effective microwave and grinding techniques. The prepared pyrazole derivatives were used as diazo disperse dyes for simultaneously dyeing and functionalization of synthetic PET fabric. The obtained results indicated that the dyed PET fabrics have very good color strength and color fastness properties for washing, perspiration, rubbing, and light. The synthesized diazo disperse dyes acquired PET fabrics with excellent UV protection. Moreover, dyed PET fabrics showed high antimicrobial activity against different strains of micro-organisms. This study has some potential limitations such as considering a quantitative comparison with commercial dyes. Also, studying antimicrobial-dye concentration relationship would enhance the precision of the performance of these synthesized dyes. We believe that taking advantages of the new and recent dyeing techniques for example, supercritical carbon dioxide, atomic layer deposition and D5 non-aqueous media dyeing system will enhance dyeing efficiency and minimize or eliminate the pollution and keep the environment clean. Consequently, the synthesized pyrazole diazo derivatives may be utilized to prepare disperse dyes for the functional dyeing of PET fabrics which could be useful for the development of protective/multifunctional clothing with aesthetic appearance.

## Data Availability

Data will be made available on request.
